# Supplementary material for: Inducing controlled cell cycle arrest and re-entry during asexual proliferation of Plasmodium falciparum malaria parasites
Source: Sci Rep. 2018 Nov 8;8:16581. doi: 10.1038/s41598-018-34964-w (PMC6224408; doi:10.1038/s41598-018-34964-w)
Supplement: Supplementary file 1 — Supplementary information [file 41598_2018_34964_MOESM1_ESM.docx]

**Inducing controlled cell cycle arrest and re-entry during asexual proliferation of *Plasmodium falciparum* malaria parasites*.***

Riëtte van Biljon^1#^, Jandeli Niemand^1#^, Roelof van Wyk^1^, Katherine Clark^1^, Bianca Verlinden^1^, Clarissa Abrie^1^, Hilde von Grüning^1^, Werner Smidt^2^, Annél Smit^1^, Janette Reader^1^, Heather Painter^3^, Manuel Llinás^3,4^, Christian Doerig^5^ and Lyn-Marié Birkholtz^1*^

^1^ Institute for Sustainable Malaria Control and ^2^ Centre for Bioinformatics and Computational Biology, Department of Biochemistry, Genetics and Microbiology, University of Pretoria, Private Bag x20, Hatfield 0028, South Africa

^3^ Department of Biochemistry & Molecular Biology and Centre for Malaria Research, Pennsylvania State University, University Park, PA 16802, USA

^4^ Department of Chemistry, Pennsylvania State University, University Park, PA 16802, USA

^5^ Infection and Immunity Program, Monash Biomedicine Discovery Institute and Department of Microbiology, Monash University, Victoria 3800, Australia

# These authors contributed equally to this work

^*^ Corresponding author Tel.: +27 12 420 2479; fax: +27 12 362 5302. E-mail address:

[lbirkholtz@up.ac.za](mailto:lbirkholtz@up.ac.za)

**Supplementary information**

**Table of Contents:**

1. **Supplementary Methods**
2. **Supplementary Figures**
3. **Supplementary Files**
4. **Supplementary methods**

### *In vitro* cultivation of intraerythrocytic *P. falciparum* parasites

*P. falciparum* (3D7 and NF54) parasites were maintained with shaking at 37°C at 5% haematocrit (human erythrocytes) in complete culture medium [RPMI 1640 medium (Sigma-Aldrich) supplemented with 25 mM HEPES (Sigma-Aldrich), 11 mM D-glucose (Sigma-Aldrich), 200 μM hypoxanthine (Sigma-Aldrich), 0.2% (w/v) sodium bicarbonate, 24 μg/ml Gentamycin (Sigma-Aldrich) and 0.5% (w/v) AlbuMAX II] under hypoxic conditions (90% N_2_, 5% O_2_, and 5% CO_2_). Parasite development was monitored with light microscopy through Giemsa staining.

**Gating for flow cytometric measurement of nucleic content**

Primary gating was performed based on background fluorescent signal from DNA-free uninfected erythrocytes to obtain the parasite-infected erythrocyte population as an indication of parasitaemia. Secondary gating was performed on the parasite-infected erythrocyte population within runs and applied to biological replicates (with technical duplicates), to segregate parasitized erythrocytes by nucleic content with 1 nucleus (1N DNA copy number) corresponding to either ring or early trophozoite forms separated from those corresponding to parasitized erythrocytes containing multiple nuclei (2N, mature trophozoite forms or >2N corresponding to schizont parasites).

**Support vector machine construction**

The SVM was trained using a linear kernel with the 3705 genes as input data together with the 30 classes. The weights of each gene's contribution to the classification of each class was calculated and the top 1% of the genes were chosen for each of the 30 classes and agglomerated into a single set of 642 genes. A non-parametric correlation coefficient (Kendall's Tau) between all classes was calculated using representative gene expression levels (maximum divergence from the control) of each class. The resultant 30x30 matrix was scaled to two dimensions using the cmdscale function in R. The data points were clustered using k-means bagged clustering to determine the optimal number of clusters.

**Experimental setup for qPCR**

RNA samples were taken from control parasites, parasites treated with 2 mM DFMO for 24 h (arrested 24h) or 30 h (arrested 30h) and parasites reversed with putrescine (24 h DFMO treatment followed by 6 h after putrescine reversal, re-entry2). A reference gene standard curve was constructed using primers specific for *P. falciparum* seryl-tRNA synthetase (Pf3D7_0717700, IDT, USA) (forward primer sequence 5’-TTCGGCAGATTCTTCCATAA-3’, and reverse primer sequence 5’-AAGTAGGAGGTCATCGTGGTT-3’). The primers used for each of the differentially expressed genes were: *pf3d7_0217500* (*cdpk1),* forward: 5’-GCTCAAGCAGCCATATTATTTATTG-3’, reverse: 5’-AAAGCTCCTAAGTATGTTGTATC-3’, *pf3d7_1356900* (*pk5),* forward: 5’-TATTTCCAGGGGTATCTG-3’, reverse: 5’-GGTAAAGGTTCATATACGGTA-3’, *pf3d7_1227500,* forward: 5’-AGAAAGAGGGAGAAAAGAGTC-3’, reverse: 5’-TCTGCACCAGGTGTTGTATC-3’, *pf3d7_1228300* (*nek1*)*,* forward: 5’-GGTGCTTACAACAATCATAATAC-3’, reverse: 5’-ATTCCTTATATTCCCTTGATACTTA-3’, *pf3d7_0617900* (*histone 3.*3), forward: 5’-CCCCAAGAAAACAACTCGC-3’, reverse: 5’-AAAGCAACAGTTCCTGGACGAT-3’. Real time quantitative PCR (qPCR) was conducted in white 384 well plates and analysed using the Roche Lightcycler 480 (Roche Diagnostics, Switzerland). Each reaction contained 5 pmol of each primer and Master Mix 2X optimised for the Roche Lightcycler 480 from the Kapa SYBRFAST qPCR kit (Kapa Biosystems, USA). The reaction was run from 1 ng cDNA for 45 cycles with a 5 min pre-incubation at 94°C. Each cycle consisted of denaturation at 94°C for 10 sec, annealing at 55°C for 20 s and extension at 68°C for 4 s, during which the amplification was measured. The amplification cycles were followed by the melt curves, consisting of 30 s incubation at 95°C, 30 s incubation at 55°C and continuous measurement until the sample reached 95°C again. For relative quantification the 2^-ΔCt^ method was used to calculate of the difference in expression expression of the gene of interest compared to the reference gene. Data were subsequently expressed as log_2_FC (arrested 24h /Control or re-entry2/ arrested 30 h).

1. **Supplementary Figures**

**a b**

**
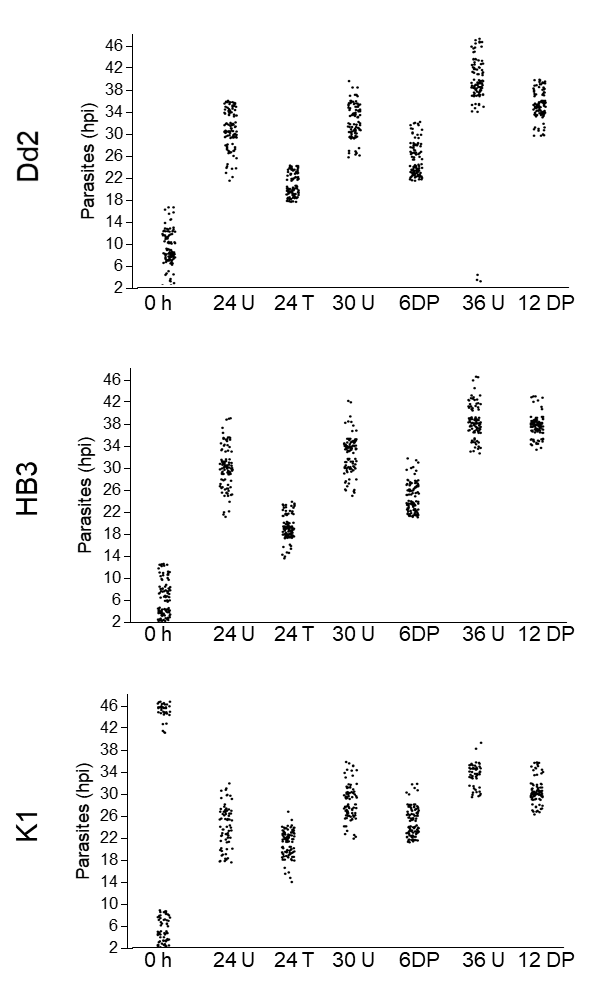
**
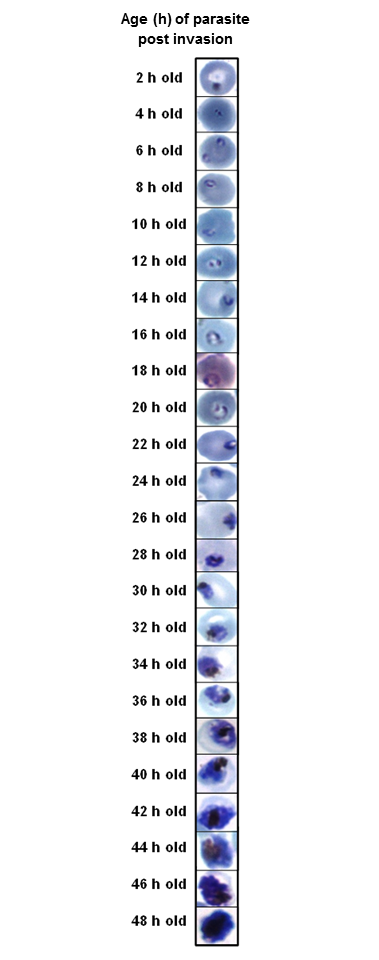


**Fig. S1 related to Fig. 2: (a)** Time course of a

synchronised, newly invasive culture (obtained with three successive rounds of sorbitol synchronisation of ring stages and subsequent magnetic purification of mature stages) monitored 2-hourly. 3D7 parasites were monitored from invasion of erythrocytes by merozoites and 2-hourly thereafter for a complete life cycle. Parasites were stained with Giemsa and viewed under 100x objective. Photos are representatives of a minimum of 100 parasites viewed at each time point. **(b)** Control (U), DFMO synchronisation (T) and reversal (DP) on different parasite strains, Dd2, HB3 and K1.

**a**

**b**

**
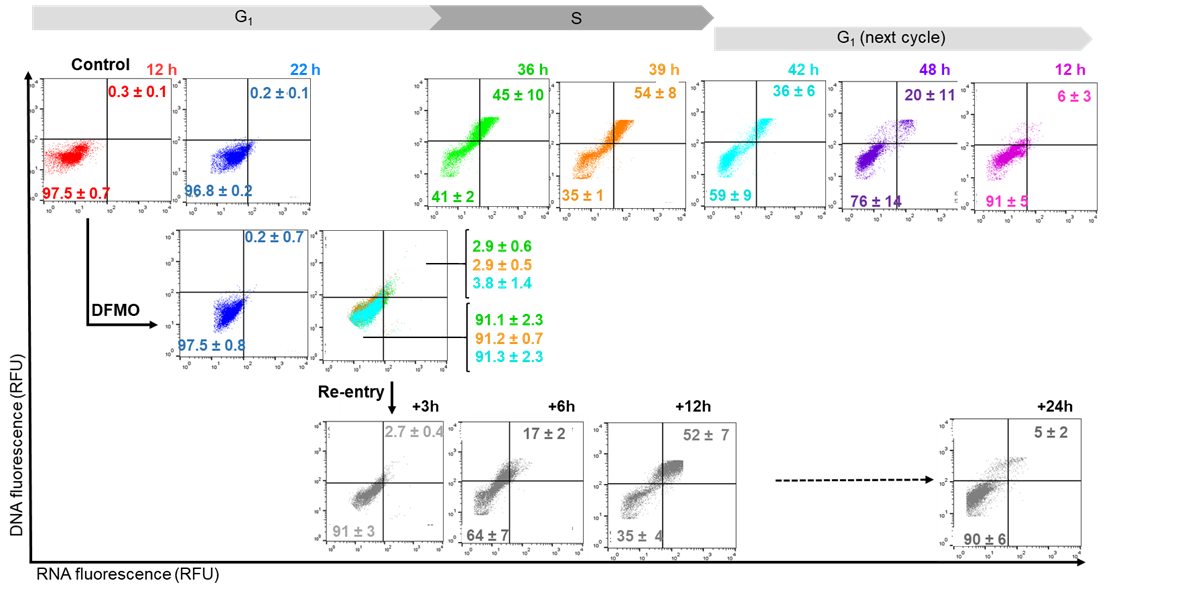
**

**Fig. S2 related to Fig 3: Reversible cell cycle arrest occurs at G_1_/S phase.** Intraerythrocytic *P. falciparum* 3D7 parasites were categorised into different cell cycle phases according to DNA and RNA content as described (Grimberg 2008)**. (a)** Gating strategy used to bin parasites based on DNA and RNA content as either in G1 or S phases of their cell cycle. **(b)** Ring-stage parasites (1% haematocrit, 10% parasitaemia) were treated with DFMO (IC_90_) or left untreated for control and samples taken at 12 hpi (red), 22 hpi (blue), 36 hpi (green), 39 hpi (orange), 42 hpi (cyan), 48 hpi (purple) and 12 hpi of the next cycle (pink). Additionally, following 24 h of DFMO treatment, putrescine (2 mM) was added to stimulate cell cycle re-entry, and samples taken 3, 6, 12 and 24 h after reversal (grayscale). The nucleic acid content of parasitized erythrocytes was determined by consecutive staining with SYBR Green I (DNA) and PyroninY (RNA); detected in the FITC or PE channel, respectively, on a Becton Dickenson FACSAria with >10 000 parasitized erythrocyte events analysed using FlowJo version X.0.7. Compensation values were calculated using single stained samples. Representative cytometric profiles are shown, with averaged quadrant data corresponding to parasite populations undergoing S-phase (upper right) and G_1_-phase (lower left) from three independent biological replicates, performed in technical duplicates, ±S.E.

Grimberg, B. T., Erickson, J. J., Sramkoski, R. M., Jacobberger, J. W. & Zimmerman, P. A. Monitoring *Plasmodium falciparum* growth and development by UV flow cytometry using an optimized Hoechst-thiazole orange staining strategy. Cytometry A 73, 546-554, doi:10.1002/cyto.a.20541 (2008).

**Fig. S3.** *P. falciparum* parasite transcriptomes were matched to cell cycle associated transcripts from non-quiescent and quiescent yeast transcriptomes (Friedlander 2006, Aragon 2006) ([www.yeastgenome.org)](http://www.yeastgenome.org)), or *P. berghei* parasites (Hall 2005) clustered according to defined cell cycle compartments. The total number of matched (*P. falciparum* to yeast/*P. berghei*) genes in each cell cycle compartment is indicated, together with the number of DE genes in italics (log_2_ fold change of >0.5 in either direction). PlasmoDB identifiers of selected transcripts: eIF4A (PF3D7_1468700); eIF4E (PF3D7_0315100), PK5 (PF3D7_1356900); RAD54 (PF3D7_0803400), MCM3 (PF3D7_0527000); MCM4 (PF3D7_1317100); MCM5 (PF3D7_1211700), MCM6 (PF3D7_1355100), MCM7 (PF3D7_0705400); ORC2 (PF3D7_0705300); DNA polymerase subunit alpha (PF3D7_0411900); DNA primase (PF3D7_0910900), proliferating cell nuclear antigen (PF3D7_1361900); nuclear complex protein 2 (PF3D7_1340300).

Friedlander, G. *et al.* Modulation of the transcription regulatory program in yeast cells committed to sporulation. *Genome Biol* **7**, R20, doi:10.1186/gb-2006-7-3-r20 (2006).

Aragon, A. D., Quinones, G. A., Thomas, E. V., Roy, S. & Werner-Washburne, M. Release of extraction-resistant mRNA in stationary phase *Saccharomyces cerevisiae* produces a massive increase in transcript abundance in response to stress. *Genome Biol* **7**, R9, doi:10.1186/gb-2006-7-2-r9 (2006).

Hall, N. *et al.* A comprehensive survey of the *Plasmodium* life cycle by genomic, transcriptomic, and proteomic analyses. *Science* **307**, 82-86, doi:10.1126/science.1103717 (2005).

**Fig. S4 related to Fig. 4 and Fig. 6: SYBR Green I qPCR confirmation of differentially expressed genes .** Gene expression is indicated as log_2_FC: for the 24 h timepoint between arrested 24 h/Control and for the 30 h timepoint re-entry 2/ arrested 30 h.

**Fig. S5 related to Fig 6A and B: Analysis of cell cycle molecular descriptors and regulators.** The transcriptomes of cell cycle arrested *P. falciparum* parasites (Arrested) as well as parasites that re-entered their cell cycles (RE1-3) were analysed in context of genes matching key terms associated with cell cycle regulation using PlasmoDB (v33). The DE genes in each functional cluster are indicated, together with transcripts showing a strong differential response (SR: log_2_ fold change of >1 in either direction) as well as genes that were refractory to KO in *P. berghei* (*P. b*) or *P. falciparum* (*P. f*) indicated with black blocks. Within the functional clusters, the expression profiles of genes of interest that were DE in RE1 (light grey block), RE2 (grey block) and RE3 (dark grey block) were highlighted in line graphs.

**Fig. S6 related to Fig. 6C. Molecular mechanisms controlling cell cycle re-entry.** A gene association network was constructed between putative regulators of cell cycle re-entry by combining co-expression analysis (GRENITS, probability linkage score >0.25) shown as solid grey edges and functional association between genes (STRING v 10.0, combined probability score >0.8) shown as dashed black edges, while associations stemming from both analyses are shown as solid black lines. Transcripts that were differentially expressed by RE1 (■), RE2 (●) and RE3 (♦) are indicated according to their increased (red) or decreased (blue) transcript abundance. Genes of interest are indicated by gene symbol on the network, (RR=ribonucleoside-diphosphate reductase large (l) subunit, PF3D7_1437200 and small subunit (s), PF3D7_1015800, ApiAP2 (1)=PF3D7_0613800, ApiAP2 (2)=PF3D7_0802100) while genes that were not subsequently analysed were collapsed into pie diagrams in terms of their functional cluster as indicated by legend.

1. **Supplementary Files**

Supplementary File S1 – Complete transcriptome dataset

Supplementary File S2 – Functional clusters

Supplementary File S3 – Cell cycle compartment analysis

Supplementary File S4 – Gene association network
